# Melatonin Preserves Blood-Brain Barrier Integrity and Permeability via Matrix Metalloproteinase-9 Inhibition

**DOI:** 10.1371/journal.pone.0154427

**Published:** 2016-05-06

**Authors:** Himakarnika Alluri, Rickesha L. Wilson, Chinchusha Anasooya Shaji, Katie Wiggins-Dohlvik, Savan Patel, Yang Liu, Xu Peng, Madhava R. Beeram, Matthew L. Davis, Jason H. Huang, Binu Tharakan

**Affiliations:** 1 Department of Surgery, Baylor Scott & White Health/Texas A&M University Health Science Center College of Medicine, Temple, Texas, United States of America; 2 Department of Medical Physiology, Texas A&M University Health Science Center College of Medicine, Temple, Texas, United States of America; 3 Department of Pediatrics, Baylor Scott & White Health/Texas A&M University Health Science Center College of Medicine, Temple, Texas, United States of America; 4 Department of Neurosurgery, Baylor Scott & White Health/ Texas A&M University Health Science Center College of Medicine, Temple, Texas, United States of America; Georgia Regents University, UNITED STATES

## Abstract

Microvascular hyperpermeability that occurs at the level of the blood-brain barrier (BBB) often leads to vasogenic brain edema and elevated intracranial pressure following traumatic brain injury (TBI). At a cellular level, tight junction proteins (TJPs) between neighboring endothelial cells maintain the integrity of the BBB via TJ associated proteins particularly, zonula occludens-1 (ZO-1) that binds to the transmembrane TJPs and actin cytoskeleton intracellularly. The pro-inflammatory cytokine, interleukin-1β (IL-1β) as well as the proteolytic enzymes, matrix metalloproteinase-9 (MMP-9) are key mediators of trauma-associated brain edema. Recent studies indicate that melatonin a pineal hormone directly binds to MMP-9 and also might act as its endogenous inhibitor. We hypothesized that melatonin treatment will provide protection against TBI-induced BBB hyperpermeability via MMP-9 inhibition. Rat brain microvascular endothelial cells grown as monolayers were used as an *in vitro* model of the BBB and a mouse model of TBI using a controlled cortical impactor was used for all *in vivo* studies. IL-1β (10 ng/mL; 2 hours)-induced endothelial monolayer hyperpermeability was significantly attenuated by melatonin (10 μg/mL; 1 hour), GM6001 (broad spectrum MMP inhibitor; 10 μM; 1 hour), MMP-9 inhibitor-1 (MMP-9 specific inhibitor; 5 nM; 1 hour) or MMP-9 siRNA transfection (48 hours) *in vitro*. Melatonin and MMP-9 inhibitor-1 pretreatment attenuated IL-1β-induced MMP-9 activity, loss of ZO-1 junctional integrity and *f*-actin stress fiber formation. IL-1β treatment neither affected ZO-1 protein or mRNA expression or cell viability. Acute melatonin treatment attenuated BBB hyperpermeability in a mouse controlled cortical impact model of TBI *in vivo*. In conclusion, one of the protective effects of melatonin against BBB hyperpermeability occurs due to enhanced BBB integrity via MMP-9 inhibition. In addition, acute melatonin treatment provides protection against BBB hyperpermeability in a mouse model of TBI indicating its potential as a therapeutic agent for brain edema when established in humans.

## Introduction

Microvascular hyperpermeability is the abnormal extravasation of plasma proteins and fluid into extravascular space and often results in vasogenic edema. Hyperpermeability in the brain occurs at the level of the blood-brain barrier (BBB). The BBB is a semipermeable membrane that separates the systemic circulation from the brain parenchyma and its optimal functioning is crucial for the maintenance of brain homeostasis. The key structural and functional elements that maintain the integrity of the BBB are the tight junctions between the neighboring endothelial cells [1]. Tight junctions primarily consists of transmembrane (occludin, claudins, junctional adhesion molecules etc.) and membrane-bound (zonula occludens [ZO]) tight junction proteins.

Zonula occludens-1 (ZO-1) acts as a scaffolding molecule mediating the link between the transmembrane tight junctions and the actin cytoskeleton [1]. It plays an important role in maintaining the important properties of the BBB including resistance and permeability [1]. Alterations in BBB integrity leading to hyperpermeability and vasogenic edema often occur following inflammation [2,3].

A variety of pro-inflammatory molecules are activated following various vascular disorders associated with traumatic brain injury (TBI), ischemia, cerebral infections, stroke, brain diseases, etc [4,5]; leading to various ill-pathologies like microvascular leakage, brain edema, neuronal injury and death [3,6]. Interleukin-1 beta (IL-1β) plays a central role in mediating the process of neuroinflammation in a variety of pathologies [7]. It is a well-studied pro-inflammatory cytokine known to induce hyperpermeability in brain endothelial cell monolayers [3,5,8,9]. It also plays an important role in TBI pathophysiology [5].

Matrix metalloproteinases (MMPs) are calcium-dependent zinc-containing proteases that play important roles in the pathophysiology of a variety of diseases. Matrix metalloproteinase-9 (MMP-9) activity and IL-1β levels increase in brain following TBI; also, the role of MMP-9 in BBB tight junction dysfunction is known [10–12]. Studies done by Wu et al (2010) in intracerebral hemorrhagic (ICH) models suggest that IL-1β may be a key mediator of MMP-9 activation and subsequent disruption of ZO-1 [13]. So far, there are no evidences that confirm the direct contribution of MMP-9 in mediating IL-1β-induced BBB hyperpermeability, although other pro-inflammatory molecules like tumor necrosis factor-alpha (TNF-α) are shown to induce MMP-9 activity and endothelial hyperpermeability [14]. Hence, we hypothesized that IL-1β treatment-induced BBB hyperpermeability may occur via MMP-9-mediated mechanisms.

Our studies further explored the role of melatonin as a potential MMP-9 inhibitor apart from being a pineal hormone with anti-inflammatory and anti-oxidant properties, based on the recent studies done by Rudra et al (2013), which indicate that melatonin inhibits MMP-9 by binding to its active catalytic site [15]. Recent studies from our laboratory demonstrate the anti-MMP-9 properties of melatonin following burn injury-induced endothelial hyperpermeability [16]. However, the role of melatonin in regulating IL-1β-induced BBB hyperpermeability, particularly the involvement of the BBB tight junctions or its effect on TBI-induced BBB hyperpermeability in a mouse model of controlled cortical impact is not known. This study aims to address the following questions:

Effect of acute IL-1β on BBB endothelial cell hyperpermeability *in vitro*.

- Comparative effects of melatonin and MMP-9 inhibitors on IL-1β-induced BBB hyperpermeability, loss of tight junctional integrity, changes in actin cytoskeletal assembly, MMP-9 activity, cell viability and ZO-1 protein/gene expression *in vitro*.
- Protective effects of melatonin against BBB breakdown induced by TBI in a mouse controlled cortical impact model of TBI *in vivo*.

## Materials and Methods

### Materials

Rat Brain Microvascular Endothelial Cells (RBMECs) and RBMEC Medium were obtained from Cell Applications Inc. (San Diego, CA). SensoLyte® 520 MMP-9 fluorometric Assay Kit was purchased from Anaspec Inc. (San Jose, CA). Transwell®-well plates were obtained from Corning Costar (New York, USA). Nunc Lab Tek II- CC, 8-well glass chamber slides, Interleukin-1β human, melatonin, fibronectin from bovine plasma, β-actin, albumin from bovine serum, Evans blue, trichloroacetic acid and fluorescein isothiocyanate-dextran-10 kDa were purchased from Sigma Aldrich (St. Louis, MO). Rabbit anti ZO-1 (Cat # 617300), mouse anti ZO-1 (Cat # 339100), 0.25% Trypsin (1X), Opti-MEM (1X)/reduced serum medium, Dulbecco's modified Eagle’s medium (DMEM; with high glucose, HEPES, no phenol red (1X)), NuPAGE Novex® 10% Bis-Tris protein gels, NuPAGE® MOPS SDS Running Buffer, NuPAGE® Transfer Buffer, HyClone Dulbecco's phosphate buffered saline (PBS, without calcium, magnesium, or phenol red), TRIzol® Reagent, SuperScript® IV First-Strand Synthesis System, Halt® Protease Inhibitor Cocktail (100X), Pierce™ ECL Western Blotting substrate and rhodamine phalloidin were purchased from Thermo Fisher Scientific (Carlsbad, CA). Anti-MMP-9 antibody (cat # ab-3) was obtained from EMD Millipore (Billerica, MA). Goat anti-mouse IgG-HRP and donkey anti-rabbit IgG-FITC secondary antibodies were purchased from Santa Cruz Biotechnology, Inc. (Santa Cruz, CA). EZViable™ Calcein AM Cell Viability fluorometric assay kit was bought from Biovision (Milpitas, CA). We also purchased Vector VECTASHIELD® Mounting Media with DAPI from Vector Laboratories (Burlingame, CA). Pierce™ BCA Protein Assay Kit and RT^2^ qPCR Primer Assay for Mouse GAPDH were purchased from Qiagen (Valencia, CA). GM6001 (also called as Ilomastat or Galardin) and MMP-9 inhibitor 1 were purchased from Calbiochem (Billerica, MA). Cell Lysis Buffer (10X) was bought from Cell Signaling Technology, Inc. (Danvers, MA). Primers were purchased from Thermo Fisher Scientific (Carlsbad, CA). MMP-9 siRNA and control siRNA (ON-TARGETplus siRNA) were purchased from Dharmacon, General Electric (Pittsburgh, PA).

### Cell culture

Primary cultures of RBMECs derived from the brain of adult Sprague Dawley rats were purchased from the Cell Applications Inc. (San Diego, CA). RBMECs were initially grown on 0.05% fibronectin-coated cell culture dishes, using the RBMEC medium in a cell culture incubator (95% O_2_, 5% CO_2_ at 37°C). Endothelial cells were treated with 0.25% trypsin-EDTA for cell detachment. Detached cells were then grown on fibronectin-coated Transwell® inserts, chamber slides or 100 mm dishes for experimental purposes. RBMEC passages 8–10 were chosen for all the experiments.

### Animals and surgeries

C57BL/6 mice (25–30 g) were purchased from Charles River Laboratories (Wilmington, MA). Animals were maintained at the Texas A&M University Health Science Center College of Medicine and Baylor Scott and White Health animal facility on a 12:12 hour dark/light cycle, with free access to food and water. The room temperature was maintained at 25° ± 2°C. Surgical and experimental procedures used in this study were conducted after approval from the Baylor Scott and White Health/Texas A&M University Health Science Center College of Medicine Institutional Animal Care and Use Committee. The facility is approved by the Association for Assessment and Accreditation of Laboratory Animal Care International in accordance with the National Institutes of Health guidelines. The animals were anesthetized with urethane, i.p. injection (2 mL/kg body weight) and was continuously observed by an investigator till the end of the study (up to one hour following traumatic brain injury). Our studies show that at this dose of urethane, animals are under deep anesthesia and has minimized any animal suffering. One-hour post-injury, animals were transcardially perfused with sterile saline containing heparin (1000 U/mL) for at least 20 minutes. This was not a survival study and no unexpected animal death was observed. The details of surgical procedures and the associated studies are described in detail in various sections below:

#### Craniotomy Procedure

The head of the animal was shaven and the surgical site on the surface of the head was cleaned with an alcohol wipe. Lubricating ointment was applied to the eyes. Midline incision on the scalp helps to remove the skin from top of the skull exposing the sagittal suture, bregma and lambda. A circular craniotomy window, 3–4 mm in diameter was made on ipsilateral hemisphere, between lambda and bregma using a microdrill. The resulting bone flap was removed. Sham animals received only craniotomy surgery, while TBI injury group received brain injury via controlled cortical impactor following craniotomy procedure.

#### Controlled cortical impactor and TBI

These studies employ Benchmark™ Stereotaxic Impactor from Leica, for inflicting TBI in mice. Following craniotomy procedure, the animals were mounted on the stereotaxic frame. An impactor probe of 3 mm diameter was used to impact the exposed part of the brain. The depth of the injury was used to determine the severity of the injury. Settings for mild TBI used in this study are: 2 millimeters depth, 0.5 meters/second velocity and 100 milliseconds contact time as described in Chen et al, 2014 [17].

### Treatments

Melatonin at a dose of 10 mg/kg was used for the acute drug administration studies. This dose was chosen based on the studies done in mouse model of TBI [18]. Melatonin was administered via the tail vein along with Evans blue injection and allowed to circulate for 30 minutes prior to TBI.

### Brain endothelial monolayer permeability *in vitro*

RBMECs were grown on fibronectin-coated Transwell® inserts as monolayers for 72–96 hours and regularly checked for confluency. Monolayers were initially exposed to phenol red free DMEM for 45 minutes to an hour. DMEM treated cells were then treated with various concentrations of IL-1β (1–100 ng/mL) for 2 hours (Panel 1A) or IL-1β (10 ng/mL) treatment at various time points (1–4 hours; Panel 1B). At the end of the treatment, FITC labeled dextran-10 kDa (5 mg/mL; 30 minutes) was applied to the luminal compartment. One hundred microliters of sample was collected from the abluminal compartment at the end of 30 minutes and measured fluorometrically at 485/520 nm (Excitation/Emission) using Fluoroskan Ascent™ FL Microplate Fluorometer and Luminometer (Vantaa, Finland). These studies provided information on the minimal dose and time exposure of IL-1β to induce BBB endothelial cell hyperpermeability.

Using the above information, a separate set of experiments were conducted to study the effect of GM6001 (broad-spectrum MMP inhibitor; 10 μM; 1 hour; Panel 2A), MMP-9 inhibitor 1 (MMP-9 specific inhibitor; 5 nM; 1 hour; Panel 2B) and melatonin (10 μg/mL; 1 hour; Panel 2C) pretreatment on IL-1β (10 ng/mL; 2 hours) treatment- induced monolayer hyperpermeability. At the end of the experiment, FITC-dextran-10 kDa was added to the donor chamber and fluorescence intensity measurements were performed as described above.

Melatonin dose and time exposure were chosen from the studies done in our lab in burn trauma models [16]. MMP-9 inhibitor 1 used in these studies is a cell-permeable, potent, selective and reversible MMP-9 inhibitor with IC_50_ at 5 nM for MMP-9 inhibition. It inhibits other MMPs at concentrations higher or lower than 5 nM, hence 5 nM was specifically chosen for these studies. MMP-9 inhibitor 1 concentration chosen is also supported by the studies done in our lab to study the effect of TNF-α on BBB hyperpermeability [14]. The dose and exposure time for GM6001 were obtained from Simao et al, 2012 [19].

Untreated cells served as control. Each experiment was performed once and then repeated four or six times. Fluorescence intensity values were plotted on the Y-axis and represented as % control. Data were expressed as mean ± % SEM and statistical differences among groups were determined by one-way analysis of variance (ANOVA) followed by Bonferroni post hoc test to determine significant differences between specific groups. A value of *p*<0.05 was considered statistically significant.

### Monolayer transfection studies

RBMECs were grown on fibronectin-coated Transwell® inserts for 24 hours and treated with control siRNA or MMP-9 siRNA at a concentration of 25 nM for 48 hours. Transfection was performed according to manufacturer’s instructions. Transfected monolayers were then exposed to IL-1β (10 ng/mL; 2 hours; Fig 3) and permeability was determined based on the leakage of FITC-dextran-10 kDa (5 mg/mL; 30 minutes) leakage from the luminal to the abluminal chamber. One hundred microliters of the sample was obtained from the abluminal chamber and fluorescence intensity was measured at 485/520 nm (Excitation/Emission) using Fluoroskan Ascent™ FL Microplate Fluorometer and Luminometer.

Untreated or control siRNA transfected cells were used as control. Each experiment was performed once and then repeated four times. Fluorescence intensity was plotted on the Y-axis and represented as % control. Data were expressed as mean ± % SEM and statistical differences among groups were determined by one-way analysis of variance (ANOVA) followed by Bonferroni post hoc test to determine significant differences between specific groups. A value of *p*<0.05 was considered statistically significant. In order to determine the efficiency of knockdown, total proteins from control siRNA or MMP-9 siRNA transfected cells were subjected to PAGE and immunoblot analysis of MMP-9 protein followed by its quantitative measurement by using ImageJ software.

### Measurement of MMP-9 activity

A SensoLyte® 520 MMP-9 fluorometric Assay Kit was employed to measure the MMP-9 activity in the cells. This kit detects the MMP-9 activity in samples by using a 5- carboxyfluorescein Ser—Leu—Gly—Arg—Lys—Ile—Gln—Ile—Gln—Lys(QXL®520)—NH2 (5-FAM/QXL™ 520 fluorescence resonance energy transfer (FRET) peptide). In intact FRET peptide, the fluorescence of 5-FAM is quenched by the QXL™ 520. However, on cleavage of the peptide by MMP-9 fluorescence is recovered and measured at 490/520 nm (Excitation/Emission).

In this procedure, RBMECs were grown in petri dishes until confluency is achieved. Cells were then pretreated with MMP-9 inhibitor 1 (5 nM; 1 hour; Panel 4A) or melatonin (10 μg/mL; 1 hour) followed by IL-1β (10 ng/mL; 2 hours; Panel 4B). At the end of the experiment, cells were washed twice in PBS and exposed to the assay buffer provided in the kit. Cells were then scraped and the cell lysates were collected. Cell lysates were briefly sonicated and centrifuged in order to collect supernatants. Supernatants were used to measure the MMP-9 activity in the cells. Equal amounts of proteins were taken in each well and 4-aminophenylmercuric acetate (APMA) was added to samples and incubated for 2 hours in dark; in order to activate the pro-MMPs. To the activated samples, MMP-9 substrate, 5-FAM/QXL 520 FRET peptide was added and incubated for another 30 minutes in dark and read fluorometrically at 490/520 nm (Excitation/Emission).

Untreated cells served as control. Each experiment was performed once and then repeated four times. MMP-9 activity was expressed as relative fluorescence units (RFU) and plotted on the Y- axis. Data were expressed as mean ± SEM and statistical differences among groups were determined by one-way analysis of variance (ANOVA) followed by Bonferroni post hoc test to determine significant differences between specific groups. A *p* value of <0.05 was considered statistically significant.

### ZO-1 immunofluorescence and rhodamine phalloidin labeling for *f*-actin

Zonula occludens-1 junctional localization and *f*-actin stress fibers were assessed using immunofluorescence and rhodamine phalloidin labeling techniques respectively. RBMECs were grown on the chamber slides for overnight. Cells were initially exposed to Opti-MEM/reduced serum medium, followed by pretreatment with MMP-9 inhibitor 1 (5 nM; 1 hour; Panel 5A) and melatonin (10 μg/mL; 1 hour; Panel 5B) and subsequently with IL-1β (10 ng/mL; 2 hours) treatment. Cells were then fixed in 4% paraformaldehyde in PBS for 10–15 minutes and permeabilized in 0.5% Triton-X 100 in PBS for another 10–15 minutes. Cells were blocked using 2% bovine serum albumin (BSA) in PBS for an hour at room temperature. Cells were then incubated overnight in anti-rabbit primary antibodies against ZO-1 (#617300; 1:150) in 2% BSA-PBS, followed by incubation with anti-rabbit IgG-FITC conjugated secondary antibody for an hour at room temperature. Cells were then washed and mounted using VECTASHEILD® Antifade Mounting Media with DAPI for nuclear staining.

For rhodamine phalloidin labeling, treatments were performed in the same way as for ZO-1 immunofluorescence study. Following treatments, cells were fixed, permeabilized, and blocked in 2% BSA-PBS as described earlier. Cells were then labeled with rhodamine phalloidin (1:50) in 2% BSA-PBS for 20 minutes (Fig 6, Panels A and B). Chamber slides were then washed and mounted using VECTASHIELD® Antifade Mounting Media with DAPI for nuclear staining. Cells were visualized and scanned at a single optical plane with an Olympus Fluoview 300 Confocal Microscope (Center Valley, PA), with a PLA PO 60X water immersion objective. Untreated cells served as control.

### Real Time-PCR studies

RBMECs were grown on 100 mm cell culture dishes until 80–90% confluency was reached. Cells were then pretreated with MMP-9 inhibitor 1 (5 nM; 1 hour) followed by IL-1β (10 ng/mL; 2 hours) (Fig 7; Panels A and B). Following treatments, cells were washed thrice in PBS and total RNA was then extracted using TRIzol® reagent according to the manufacturer’s instructions. RNA concentration and quality were determined by employing the ratio of absorbance at 260/280 nm using Biotek Synergy Hybrid Spectrophotometer (Winooski, VT). Reverse transcription was performed using the SuperScript® IV First-Strand Synthesis System. Quantitative real time PCR was performed using the RT^2^ SYBR Green Fluor qPCR Mastermix with the following primer pairs for ZO-1: Forward primer: 5′-CCTCTGATCATTCCACACAGTC-3′, Reverse primer: 5′-TAGACATGCGCTCTTCCTCTCT-3′, MMP-9: Forward primer: 5'- GGCTAGGCTCAGAGGTAA-3', Reverse primer: 5'-GACGTTGTGTGAGTTCCAG-3' and GAPDH: Forward primer: 5′-AATGTATCCGTTGTGGATCT-3′, Reverse primer: 5′-CAAGAAGGTGGTGAAGCAGG-3′ were used. Real-time PCR detection was carried out using Stratagene Mx3000P qPCR System, Agilent Technologies (La Jolla, CA), using 1 μL of cDNA for 10 minutes at 95°C, followed by 40 cycles of 15 sec at 95°C for denaturation and 1 min at 60°C for annealing. Relative abundances of target genes were calculated by normalizing Ct values to endogenous control glyceraldehyde 3-phosphate dehydrogenase (GAPDH).

Cells were treated with IL-1β, while untreated cells served as control. Each experiment was repeated three times. Relative gene expression of ZO-1 was obtained by normalizing the C_t_ values to the endogenous control GAPDH for each repeat. Normalized C_t_ values were expressed as mean ± SEM. Statistical differences among groups were determined by one-way analysis of variance (ANOVA) followed by Bonferroni post hoc test to determine significant differences between specific groups. A *p* value of <0.05 was considered statistically significant.

### Western blot analysis of ZO-1

Western blots were performed to determine the potential changes in ZO-1 protein expression. Cells were initially exposed to reduced serum medium followed by IL-1β (10 ng/mL; 2 hours) treatment. At the end of the study, cells were washed twice in ice-cold PBS and incubated in ice-cold cell lysis buffer (1X) along with protease inhibitor cocktail (1X) for 5 minutes in cell culture dishes. Cells were then scraped, sonicated and centrifuged at 14,000*g* for 10 minutes at 4°C. Supernatant was collected from the extracts and protein concentration was determined using protein assay kit. Equal amounts of total protein (50 μg) were separated by sodium dodecyl sulphate-polyacrylamide gel electrophoresis (SDS-PAGE) on 10% Bis-Tris precast gels at constant voltage (145 V) for 180 minutes. Proteins were then transferred onto the nitrocellulose membrane at constant voltage (30 V) for overnight and the membranes were blocked using 5% nonfat dry milk in Tris-Buffered Saline (TBS) with 0.05% Tween-20 and subsequently incubated with primary mouse monoclonal anti ZO-1 antibody (1:250 dilution). Membranes were washed thrice in TBS-T and incubated with the goat anti-mouse IgG- HRP conjugated secondary antibody. After washing, the immunoblots were visualized by ECL Western Blotting Substrate. Untreated cells served as control. Equal amount of protein sample loading was verified by assessing β-actin protein expression.

### Cell viability studies

In order to determine if the changes in permeability was due to the loss of cell viability or not, a cell viability assay was conducted. An EZViable™ Calcein AM Cell Viability Assay Kit (Fluorometric) was used for quantify the number of viable cells. Calcein AM is a non-fluorescent, hydrophobic compound that easily penetrates intact and live cells. Hydrolysis of the calcein AM by intracellular esterase produces a hydrophilic, strongly fluorescent compound that is retained in the cell cytoplasm, which can be measured at 485/530 nm (Excitation/Emission).

Equal numbers of cells were grown on sterile black 96 well trays. On reaching confluency, growth media is discarded and cells were washed in PBS and pre-exposed to phenol red-free medium for 1 hour. Cells were divided into control (untreated) and IL-1β ss(10 ng/mL) treatment groups. Hydrogen peroxide at 100 mM was used as a positive control. Following treatments, cells were then exposed to calcein buffer solution (calcein AM: calcein dilution buffer in 1:500 dilution) and incubated at 37°C for 30 minutes and a fluorometric reading was obtained.

Fluorescence intensity was plotted on the Y-axis and represented as % control. Data were expressed as mean ± % SEM and statistical differences among groups were determined by one-way analysis of variance (ANOVA) followed by Bonferroni post hoc test to determine significant differences between specific groups. A *p* value of <0.05 was considered statistically significant.

### Evans blue leakage studies

Evans blue dye binds to the albumin in the blood enabling us to detect the vascular leakage into the extravascular tissue following TBI. C57BL/6 mice (25–30 g) were anesthetized with urethane, i.p. injection (2 mL/kg body weight) followed by Evans blue dye, i.v. injection (2% wt/vol in saline; 4 mL/kg body weight). Melatonin was injected at a concentration of 10 μg per gram body weight of the animal. A stock of 50μg/μL of 100% alcohol was prepared. Melatonin from the stock concentration was then diluted in Evans blue, based on the body weight of the animal. The total alcohol concentration was less than 0.5% of the total blood volume. Evans blue was allowed to circulate in the animal for 30 minutes prior to performing sham surgery (only craniotomy) or TBI using controlled cortical impactor. Drugs were given to the animal along with Evans blue injection and allowed to circulate for 30 minutes as well. Settings for mild TBI are described in the CCI procedure.

Animals were grouped into Sham (only craniotomy; n = 6), Vehicle + Sham (75% ethanol injection followed by craniotomy; n = 6), Vehicle + TBI group (75% ethanol followed by mild TBI; n = 5), and melatonin +TBI group (melatonin [10 μg/gram body weight of the animal] injection followed by mild TBI; n = 6). The vehicle was combined with Evans Blue when given and the total alcohol concentration was less than 0.5% of the total blood volume.

One-hour post-TBI, animals were transcardially perfused with sterile saline containing heparin (1000 U/mL) for at least 20 minutes. Brains were extracted and brain cortex was carefully separated and weighed. Brain cortices were then homogenized in 1 mL of 50% (wt/vol) trichloroacetic acid (TCA) in saline. Homogenate was then centrifuged at 6,000*g* for 20 minutes at 4°C. Supernatants were extracted and further diluted in 3 parts of ethanol (1:3; 50% TCA: 95% ethanol). Samples were then quantitated fluorometrically at 620/ 680 nm (Excitation/Emission) using Biotek Synergy Hybrid H1 spectrophotometer (Winooski, VT). Evans blue concentration in the samples were evaluated using external standards for Evans blue ranging from 50–1000 ng/mL, prepared in same solvent (1:3; TCA: 95% ethanol). Evans blue amount in the samples was expressed as ng/brain cortex ± SEM. Statistical differences among groups were determined by one-way analysis of variance (ANOVA) followed by Bonferroni post hoc test to determine significant differences between specific groups. A *p* value of <0.05 was considered statistically significant.

### Statistical Analysis

Data are expressed as the mean ± % SEM for the monolayer permeability, monolayer transfection and cell viability studies; while mean ± SEM is employed for data obtained from MMP-9 activity assay, real time-PCR studies and Evans blue leakage studies. Statistical differences among groups were determined by one-way ANOVA followed by Bonferroni post hoc test to determine significant differences between specific groups. Student’s *t*-test was employed for comparing the protein expression data. *p*<0.05 was considered a statistically significant difference.

## Results

### IL-1β treatment induces BBB endothelial cell monolayer hyperpermeability

**Fig 1:**
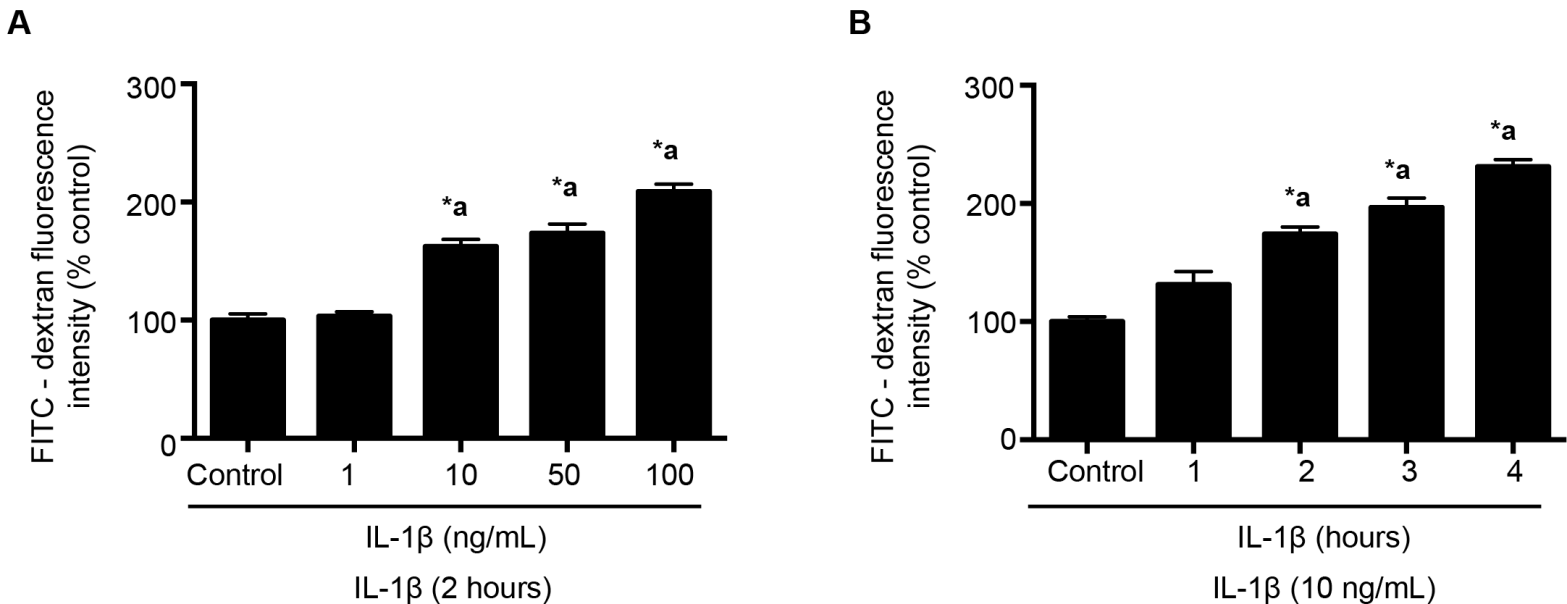
IL-1β treatment induces dose and time dependent increase in monolayer hyperpermeability. In Panel A, IL-1β treatment at doses 10, 50 and 100 ng/mL for 2 hours are shown to significantly increase BBB permeability compared to the control group (n = 4; *p*<0.05). Panel B indicates significant increase in IL-1β induced BBB permeability at 2, 3 and 4 hours compared to the control (n = 4; *p*<0.05). Monolayer permeability is expressed as a percentage control of FITC-dextran-10 kDa fluorescent intensity, plotted on the Y-axis. Data are expressed as mean ± % SEM. ‘*a’ indicates significant increase compared to the control group.

Endothelial cell monolayers were pretreated with various concentrations of IL-1β ranging from 1–100 ng/mL for a 2-hour period. Results from this study confirmed that IL-1β treatment at 10 ng/mL for 2 hours was found to be the minimal dose to induce monolayer hyperpermeability (Fig 1; Panel A; *p* <0.05).

In a separate set of experiments, RBMECs were then treated with IL-1β (10 ng/mL) for various time periods ranging from 1 to 4 hours. Result from this study further confirmed that IL-1β treatment (10 ng/mL; 2 hours) was the minimal dose to induce RBMEC monolayer hyperpermeability (Fig 1; Panel B; *p* <0.05). Hence, IL-1β treatment at 10 ng/mL dose for a 2-hour period was used to induce monolayer hyperpermeability in the following experiments.

### MMP-9 specific inhibition attenuates IL-1β-induced BBB endothelial cell hyperpermeability

**Fig 2:**
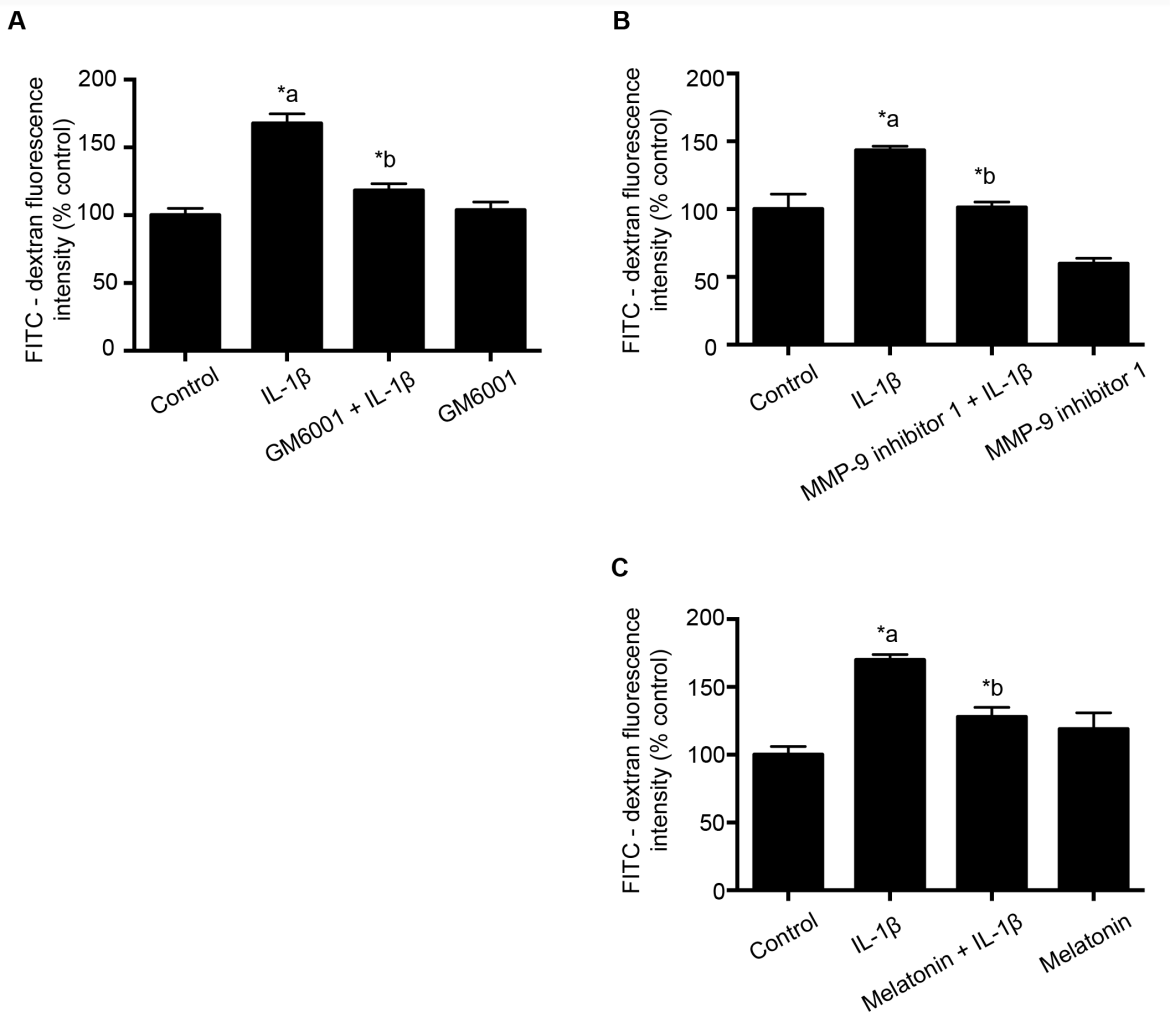
GM6001, MMP-9 inhibitor 1 and melatonin pretreatment attenuates IL-1β treatment-induced monolayer hyperpermeability. Panel A indicates the effect of GM6001 (broad-spectrum MMP inhibitor; n = 4); while Panels B and C employ MMP-9 specific inhibitors: MMP-9 inhibitor 1 (n = 4) and melatonin (n = 6) pretreatment on IL-1β (10 ng/mL; 2 hours)—induced monolayer hyperpermeability. Monolayer permeability is expressed as a percentage control of FITC-dextran-10 kDa fluorescence intensity, plotted on the Y-axis. Data are expressed as mean ± % SEM. ‘*a’ indicates significant increase compared to control group; ‘*b’ indicates significant decrease compared to the IL-1β treated group. *p*<0.05 was considered statistically significant.

Initial preliminary studies to confirm the involvement of MMPs in mediating IL-1β- induced monolayer hyperpermeability; GM6001 (broad-spectrum MMP inhibitor) was used. Endothelial cell monolayers were pretreated with GM6001 (Fig 2; Panel A; 10 μM; 1 hour) followed by IL-1β (10 ng /mL; 2 hours).

To study the effect of MMP-9 specific inhibition, MMP-9 inhibitor 1 and melatonin were employed. IL-1β (10 ng/mL; 2 hours) treatment-induced monolayer hyperpermeability was significantly attenuated on pretreatment with MMP-9 inhibitor 1 (Fig 2; Panel B; 5 nM; 1 hour) and melatonin (Fig 2; Panel C; 10 μg/mL; 1 hour). Permeability was assessed by monolayer permeability assays as described earlier.

### MMP-9 knockdown attenuates IL-1β-induced endothelial cell hyperpermeability

**Fig 3:**
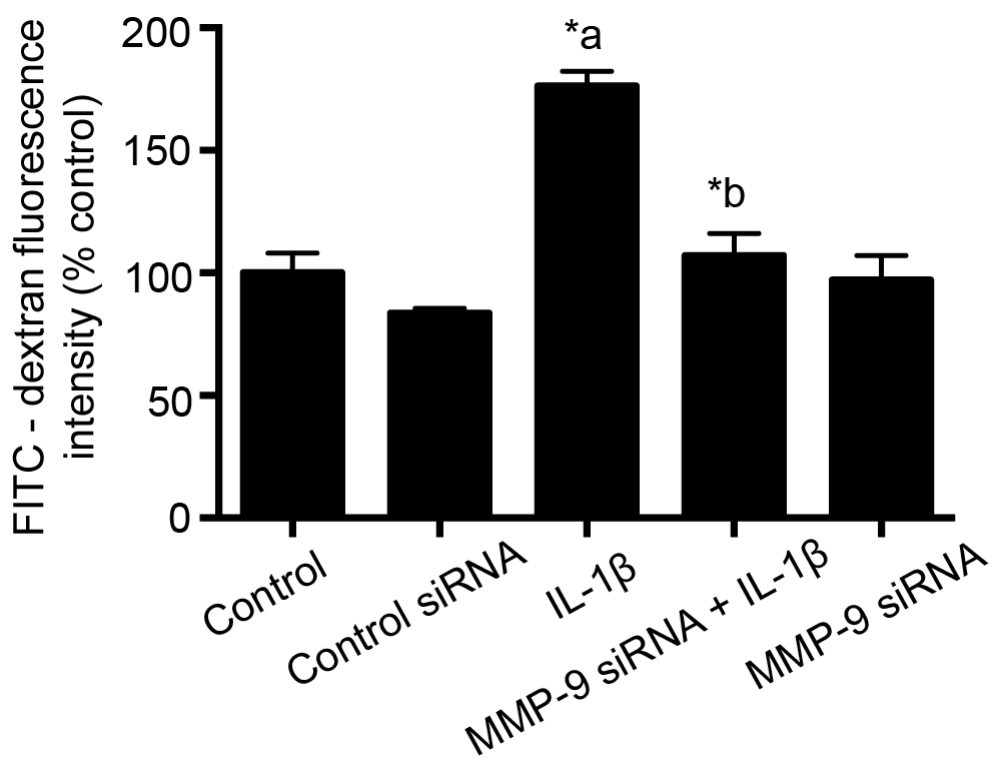
Knockdown of MMP-9 by siRNA attenuates IL-1β treatment-induced monolayer hyperpermeability. Monolayer permeability is expressed as percentage flux of FITC-dextran-10 kDa fluorescence intensity, plotted on the Y-axis. Data are expressed as mean ± % SEM. ‘*a’ indicates significant increase compared to the control group; ‘*b’ indicates significant decrease compared to the IL-1β (10 ng/mL; 2 hours) treatment group. siRNA transfected groups were compared to control siRNA transfected group (n = 4; p<0.05).

Significant contribution of MMP-9 in attenuating IL-1β-induced endothelial cell hyperpermeability is further supported by siRNA transfection studies. IL-1β (10 ng/mL; 2 hours) treatment to MMP-9 knockdown (25 nM; 48 hours) cells significantly reduced IL-1β treatment-induced monolayer hyperpermeability (Fig 3). siRNA treated groups were compared to the control siRNA group, while the IL-1β alone treated group was compared to the control group.

Immunoblot analysis of MMP-9 protein following transfection of the cells with control siRNA or MMP-9 siRNA showed a 37% decrease in MMP-9 levels. This decrease was statistically insignificant (Student’s *t*-test; S1 Fig).

### IL-1β treatment-induced MMP-9 activity is attenuated by pretreatment with MMP-9 inhibitor 1 and melatonin

**Fig 4:**
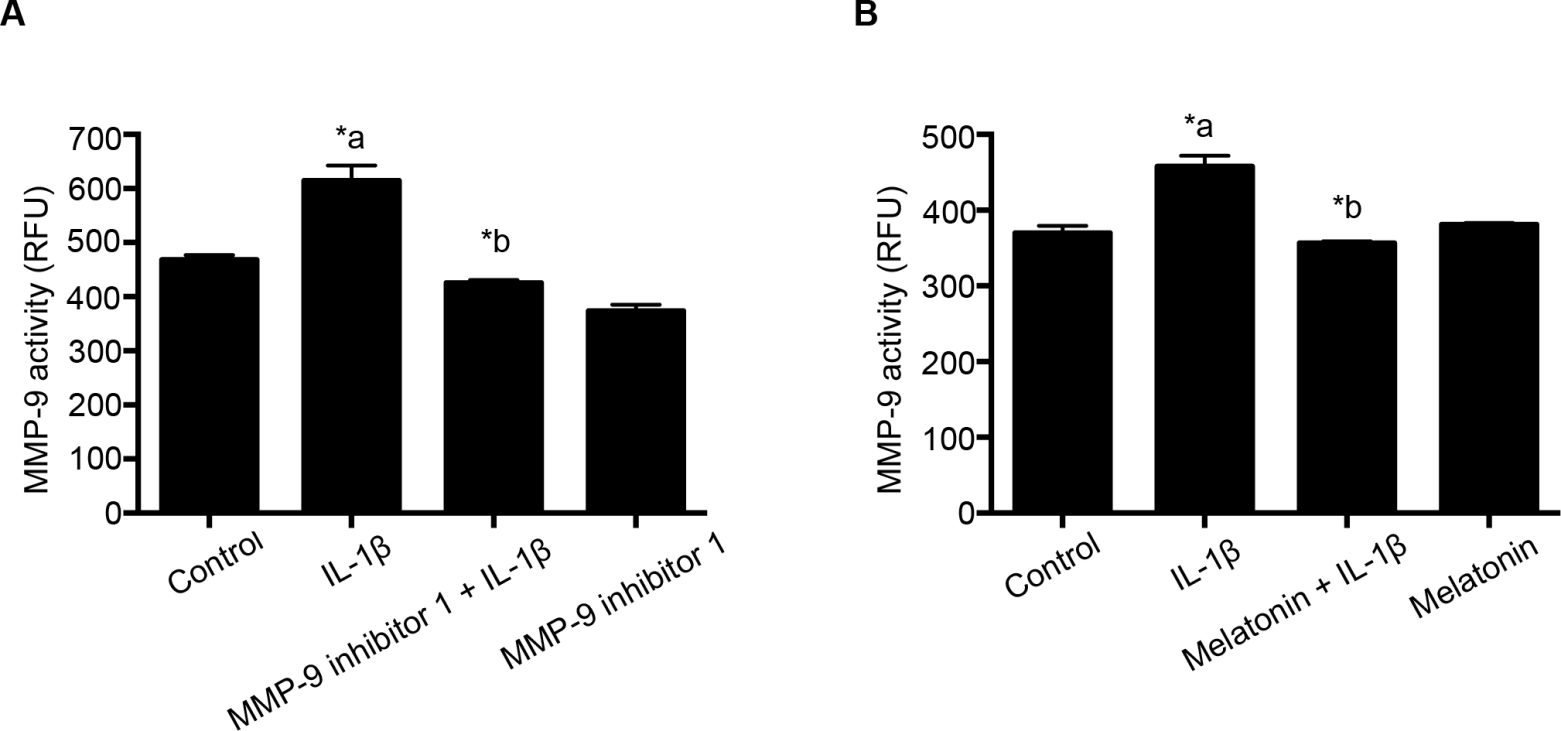
MMP-9 inhibitor 1 and melatonin pretreatment attenuates IL-1β treatment- induced MMP-9 activity. MMP-9 inhibitor 1 (n = 4) and melatonin (n = 5) pretreatment attenuated IL-1β treatment-induced MMP-9 activity in RBMECs. MMP-9 activity is expressed as relative fluorescence units (RFU), plotted on the Y-axis. Data are expressed as mean ± SEM. ‘*a’ indicates significant increase compared to the control group; ‘*b’ indicates significant decrease compared to the IL-1β (10 ng/mL; 2 hours) treatment group. *p*<0.05 was considered statistically significant.

IL-1β treatment significantly increased MMP-9 activity in RBMECs, while pretreatment with MMP-9 inhibitor 1 (5 nM; 1 hour) or melatonin (10 μg/mL; 1 hour) significantly attenuated IL-1β (10 ng/mL; 2 hours) treatment-induced MMP-9 activity (Fig 4; Panels A and B). MMP-9 activity measurements were performed as described in the methods section. MMP-9 inhibitor 1 was used as a pharmacological inhibitor of MMP-9; however, anti- MMP-9 properties of melatonin are still not as well-established. Our studies support the role of melatonin as a potential MMP-9 inhibitor.

### Melatonin and MMP-9 inhibitor 1 provides protection against IL-1β treatment- induced loss of ZO-1 junctional integrity

**Fig 5:**
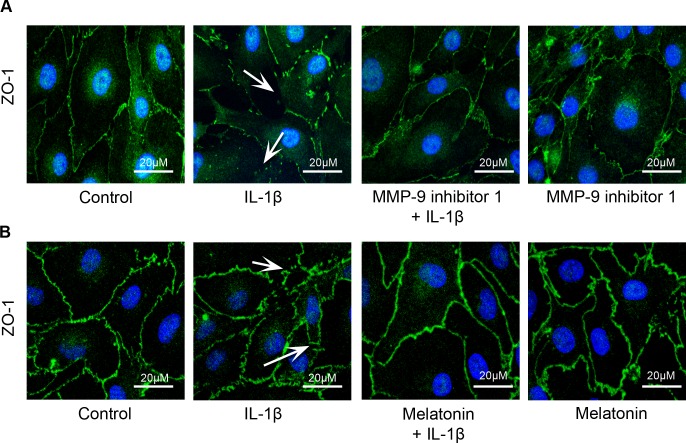
MMP-9 inhibitor 1 and melatonin pretreatment protects against IL-1β treatment-induced loss of ZO-1 junctional integrity. IL-1β (10 ng/mL; 2 hours) treatment-induced ZO-1 junctional disruption (white arrows) was decreased on pretreatment with MMP-9 inhibitor 1 (n = 4) and melatonin (n = 4).

Cells were processed for immunofluorescence localization of tight junction protein, ZO-1. IL-1β (10 ng/mL; 2 hours) treatment-induced ZO-1 junctional discontinuity (white arrows; Panel 5A and B) compared to the control cells (Fig 5). Pretreatment with MMP-9 inhibitor 1 (5 nM; 1 hour) and melatonin (10 μg/mL; 1 hour) decreased IL-1β treatment- induced ZO-1 junction integrity (Fig 5). Untreated cells served as control. These studies support that IL-1β treatment-induced monolayer hyperpermeability occurs via ZO-1 junctional disruption and possibly by ZO-1 redistribution.

### Melatonin and MMP-9 inhibitor 1 provides protection against IL-1β treatment- induced *f*-actin stress fiber formation

**Fig 6:**
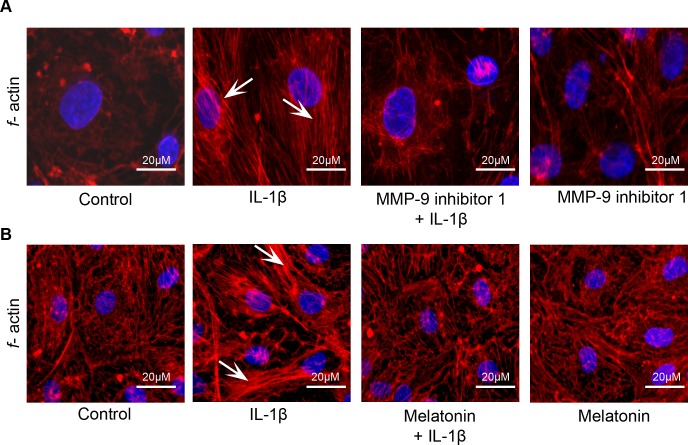
MMP-9 inhibitor 1 and melatonin pretreatment reduces IL-1β treatment- induced *f*-actin stress fiber formation. IL-1β (10 ng/mL; 2 hours) treatment-induced *f*-actin stress fiber formation (white arrows) was decreased by pretreatment with MMP-9 inhibitor 1 (n = 4) and melatonin (n = 4).

For assessing the cytoskeletal assembly, rhodamine phalloidin labeling technique was performed. Untreated cells served as control. IL-1β treatment-induced *f*-actin stress fiber formation (white arrows; Fig 6; Panels A and B) was reduced on pretreatment with MMP-9 inhibitor 1 and melatonin.

### IL-1β treatment neither induces ZO-1 mRNA expression nor alters ZO-1 protein expression

**Fig 7:**
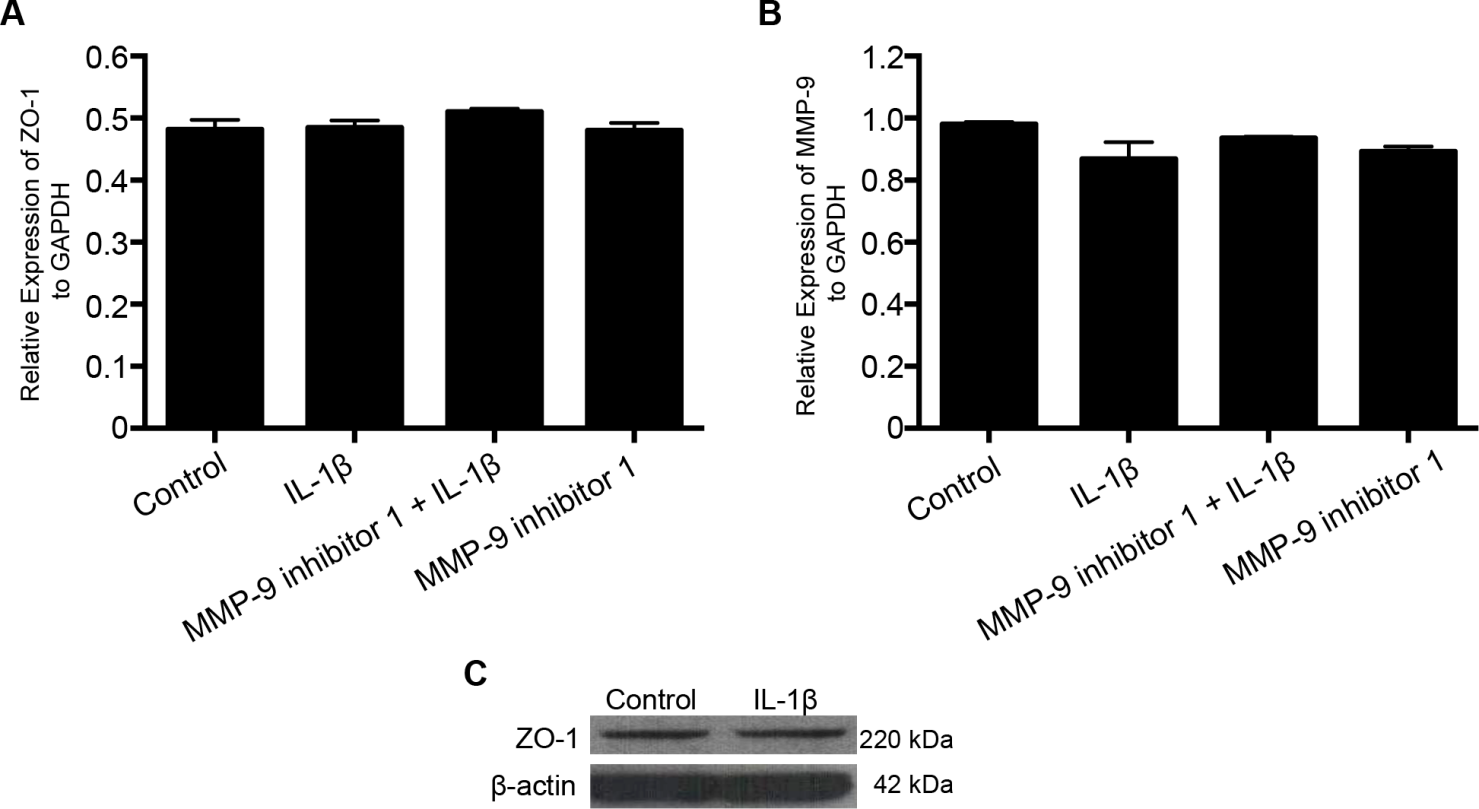
IL-1β treatment does not induce ZO-1 mRNA or protein expression. IL-1β (10 ng/mL; 2 hours) treatment neither induces ZO-1/MMP-9 mRNA expression (n = 3) nor alter ZO-1 protein expression (n = 4). RT-PCR data plotted on the Y-axis are expressed as relative expression of ZO-1 normalized to GAPDH. Data are represented as mean ± SEM.

IL-1β treatment (10 ng/mL; 2 hours) did not alter ZO-1 or MMP-9 mRNA expression by RT-PCR studies (Fig 7; Panels A and B). IL-1β treatment (10 ng/mL; 2 hours) did not the alter ZO-1 protein expression (Fig 7; Panel C).

### IL-1β treatment does not induce cell death in endothelial cells

**Fig 8:**
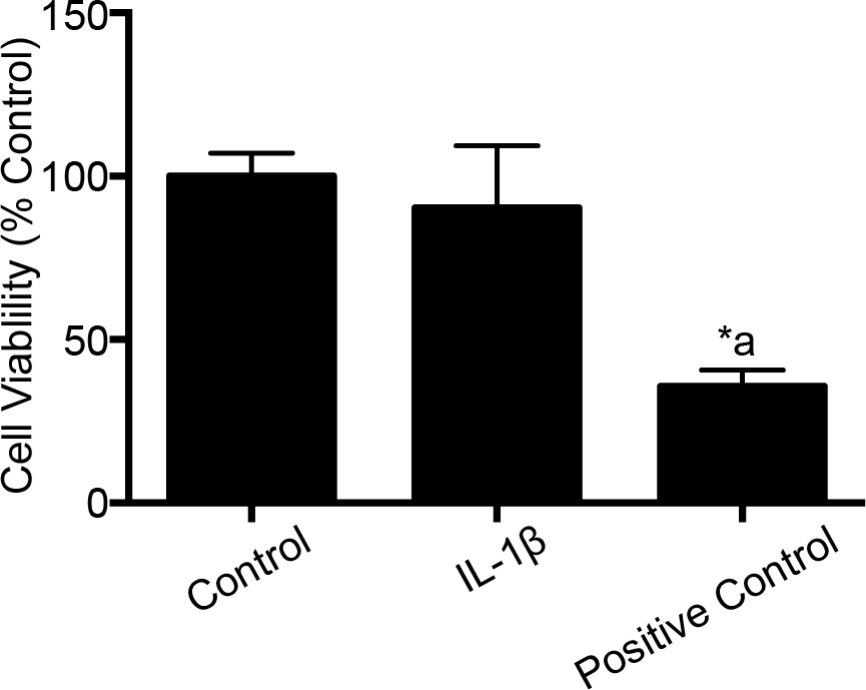
IL-1β treatment does not induce cell death. IL-1β (10 ng/mL; 2 hours) treatment had no effect on cell viability (n = 5). Hydrogen peroxide (used as a positive control) treatment decreases cell viability significantly (*p<*0.05). Data are expressed as mean ± % SEM. ‘*’ indicates statistical significance. ‘*a’ indicates significant decrease compared to the control group.

IL-1β treatment at 10 ng/mL for 2 hours did not cause any significant change in cell viability (Fig 8). Hydrogen peroxide at 100 mM concentration for 2 hours was used as a positive control. Hydrogen peroxide significantly attenuated cell viability compared to untreated control group (*p* <0.05).

### Melatonin pretreatment attenuates TBI-induced BBB hyperpermeability

**Fig 9:**
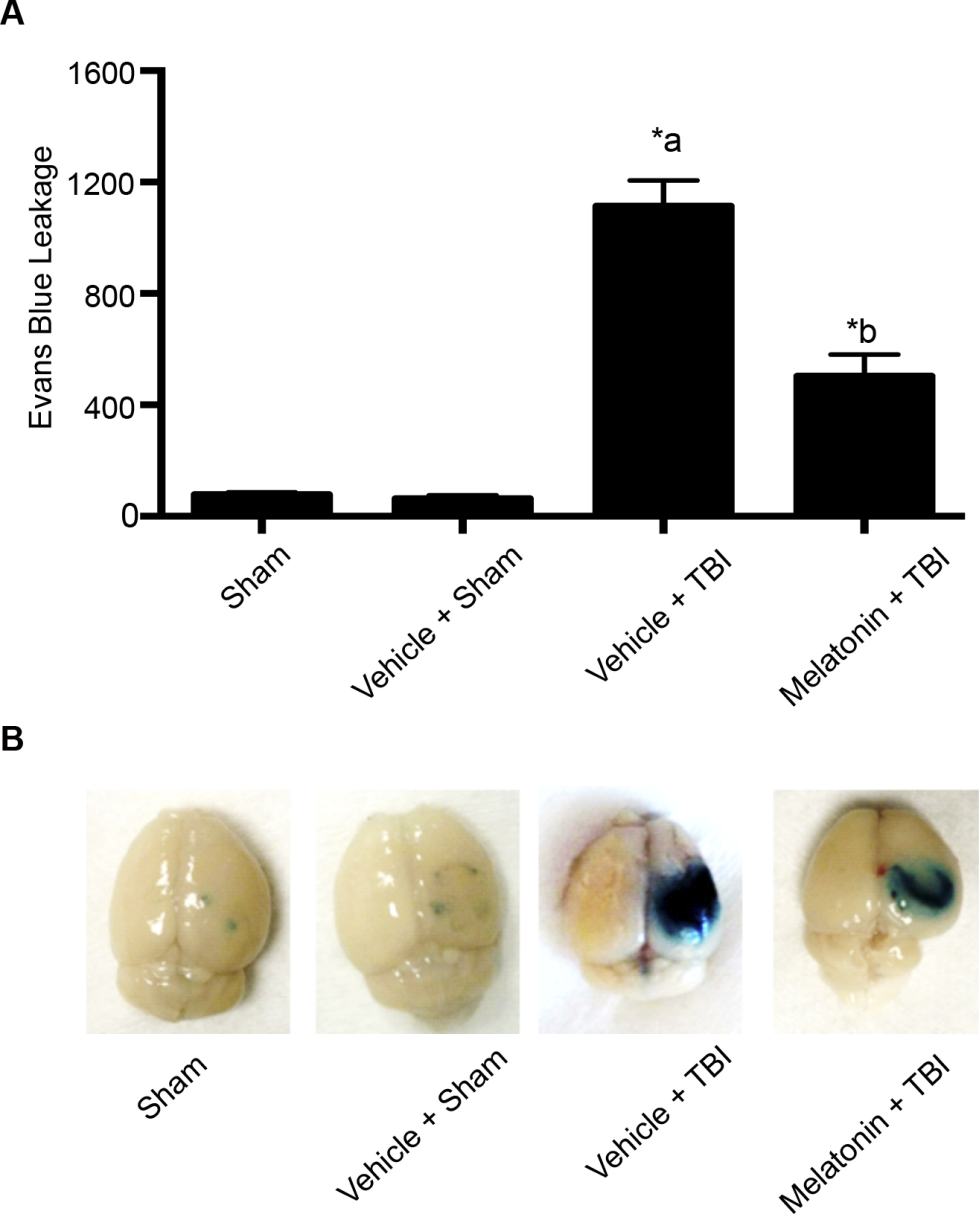
Melatonin pretreatment attenuates TBI-induced BBB hyperpermeability studied by Evans blue dye extravasation method (Panel A). Pictorial representation of the brain tissue from various groups is shown in Panel 9B. Sham injury group was used as the baseline for all comparisons. Melatonin (10 μg/gram body weight of the animal) pretreatment significantly attenuated TBI-induced Evans blue leakage into the extravascular tissue space (*p*<0.05). Animals were divided into sham (n = 6), vehicle + sham (n = 6), vehicle + TBI (n = 5) and melatonin + TBI (n = 6). Data are expressed as ng/brain cortex ± SEM. ‘*’ indicates statistical significance. ‘a’ indicates significant increase compared to the sham injury/vehicle + sham injury group and ‘b’ indicates significant decrease compared to the vehicle + TBI group.

Mice subjected to TBI demonstrated significant increase in Evans blue leakage compared to the sham animals. Evans blue extravasation was performed using ipsilateral brain cortices. Pretreatment with melatonin attenuated mild TBI-induced Evans blue leakage into the brain tissue (Fig 9; Panels A and B). Evans blue leakage was assessed fluorometrically at 620/680 nm (Excitation/Emission). This study suggests that melatonin can be used as a potential therapeutic agent in attenuating BBB hyperpermeability that occurs following TBI.

## Discussion

The results from this study demonstrate that melatonin has protective effects against IL-1β-induced BBB dysfunction and hyperpermeability *in vitro* via MMP-9 inhibition and by maintaining tight junctional and cytoskeletal integrity. It also effectively decreases acute BBB hyperpermeability in a mouse controlled cortical impact model of traumatic brain injury *in vivo*. Furthermore, the results show that IL-1β-induced acute barrier dysfunctions are not due to alterations in endothelial cell viability or a decrease in the content or expression of the key tight junction associated protein ZO-1.

Matrix metalloproteinases (MMPs) are zinc and calcium proteases that play several roles in vascular physiology and pathophysiology [16,20,21]. Matrix metalloproteinase-9 levels are found to increase significantly in the cerebrospinal fluid (CSF) samples of the traumatic brain injury patients [11]. Identification of MMP-9 in the CSF may indicate their role in BBB disruption, which is supported by its ability to degrade various tight junction proteins such as claudin-5, occludin, and zonula occludens (ZO-1) in cultured brain endothelial cells [12]. Although studies done in intracerebral hemorrhagic (ICH) models suggest that IL-1β may induce MMP-9 activation and subsequent ZO-1 disruption [13], no elaborate studies have been done to imply its significance in regulating BBB dysfunction and hyperpermeability. Our studies further employ various MMP inhibitors like GM6001, MMP-9 inhibitor 1 and melatonin, in order to study the effect of MMP-9 inhibition on IL-1β-induced BBB dysfunction and hyperpermeability.

Interleukin-1β upregulation is also observed following experimental brain injuries [13,22] and it has been attributed to several of the adverse consequences of traumatic brain injury. Our studies support the contribution of IL-1β in inducing BBB dysfunction and hyperpermeability as demonstrated in our *in vitro* permeability studies, changes in ZO-1 junctional localization, and *f*-actin stress fiber formation. Although, IL-1β treatment induced ZO-1 junctional disruption and BBB hyperpermeability, no change in the total ZO-1 protein or gene expression was observed. These studies indicate a possibility that IL-1β treatment-induced alterations in the BBB may not be due to changes at the gene or protein level but could be due to displacement of ZO-1 from the tight junctions thus detaching itself from the transmembrane tight junction as well as the actin cytoskeleton. Cytoskeletal changes in the form of increased actin stress fibers, observed in our studies following IL-1β treatment parallel the alterations in monolayer permeability and barrier dysfunctions. Recent studies demonstrate that actin stress fiber formation is not necessarily connected to increase in permeability, but might actually reflect a cellular response to repair disrupted barrier function [23,24]. Our observation thus, suggest that the barrier is undergoing repair process and the hyperpermeability observed may be reversible. The involvement of other intermediate signaling pathways that are activated by mitogen activated protein kinases like c-Jun N-terminal kinases (JNK) and p38 as described previously, are also possible [3,25].

IL-1β treatment induces MMP-9 activity in brain endothelial cells *in vitro* and one of the mechanisms by which MMP-9 inhibitor 1 and melatonin pretreatment attenuates IL-1β-induced MMP-9 activity may occur by direct binding of MMP-9 inhibitor 1 and melatonin to MMP-9. However, these studies need to be explored further in order to understand how MMP-9 inhibitors inhibit IL-1β-induced BBB hyperpermeability. As MMP-9 is indicated to activate protein kinase C via extracellular signal-regulated kinases (ERK) in TBI [26], this can be a potential mechanism that needs further studies.

Our cell culture studies employ commercially available primary cultures of microvascular endothelial cells isolated from rat brain. They are then cultured continuously for the purpose of the experiment upto 8–10 passages. Although they are most representative to the *in vivo* experimental models, they suffer from various limitations like relative short life span, susceptibility to contamination and may not also fully act like a tissue due to the complexity of the media apart from the fact that there is considerable variation in population and between preparations. These finite cell lines also tend to differentiate over a period of time and the culture tends to select for aberrant cells.

The present study employed pharmacological and endogenous MMP-9 inhibitors i.e. MMP-9 inhibitor 1 and melatonin in order to test their effect on IL-1β treatment- induced BBB dysfunction and hyperpermeability. Use of melatonin has great advantage as it can cross the BBB due to its lipophilic nature and can act as a neuroprotectant [27]; apart from the fact that is a relatively less expensive drug that is available over-the- counter with no known adverse effects. Our results underline the need for future studies to explore the therapeutic advantages of melatonin against BBB dysfunction and edema formation following TBI. Our studies, while supporting the recent observation that melatonin is protective against brain edema and elevated ICP in a rat weight drop model of TBI [28], further shows how it works in an acute setting and also its potential mechanisms of action at the level of the tight junctions of the blood-brain barrier.

Although these results support the role of melatonin in regulating BBB endothelial functions via MMP-9 inhibition, we do not believe its contribution is only owing to its MMP-9 inhibitory properties and there is a need to explore other possible mechanisms. Future studies should aim to address the effect of melatonin on 1) tissue inhibitors of MMPs (TIMPs), 2) transcription factors responsible for MMP-9 expression such as nuclear factor kappa-light-chain-enhancer of activated B cells (NF-κB) and activator protein (AP-1), as suggested by Grossetete et al (2009) [11], 3) on various ERK MAPK and 4) phosphorylation status of the tight junctions.

In conclusion, the preset study demonstrates that melatonin has protective effects against acute IL-1β-induced BBB dysfunction and hyperpermeability via MMP-9 inhibition and by preserving tight junction integrity *in vitro*. The protective effects of melatonin in an acute setting *in vitro* parallel with its protective effects against TBI in a controlled cortical impact model of TBI *in vivo* and provide great promise to early regulation of secondary injuries.

## Supporting Information

S1 Fig Immunoblot analysis of MMP-9 demonstrating an insignificant reduction in MMP-9 protein following MMP-9 siRNA transfection in RBMECs compared with control siRNA group. A. Representative bands from immunoblot analysis. B. Analysis of the data using ImageJ (Student’s *t*-test; p>0.05). The data is expressed as relative expression of MMP-9 to β-Actin.
(TIF)

## Abbreviations

BBB: blood-brain barrier
CCI: controlled cortical impactor
FITC: fluorescein isothiocyanate
DMEM: Dulbecco’s Modified Eagle Medium
GAPDH: glyceraldehyde 3-phosphate dehydrogenase
IL-1β: Interleukin-1β
MMP-9: Matrix metalloproteinase-9
PBS: phosphate buffered saline
RBMEC: rat brain microvascular endothelial cells
TJ: Tight Junctions
TBI: Traumatic Brain Injury

## References

[1] Alluri H, Wiggins-Dohlvik K, Davis ML, Huang JH, Tharakan B (2015) Blood-brain barrier dysfunction following traumatic brain injury. Metab Brain Dis 30: 1093–1104. 10.1007/s11011-015-9651-7 25624154

[2] Bolton SJ, Anthony DC, Perry VH (1998) Loss of the tight junction proteins occludin and zonula occludens-1 from cerebral vascular endothelium during neutrophil-induced blood-brain barrier breakdown in vivo. Neuroscience 86: 1245–1257. 9697130 10.1016/s0306-4522(98)00058-x

[3] Rigor RR, Beard RS Jr., Litovka OP, Yuan SY (2012) Interleukin-1beta-induced barrier dysfunction is signaled through PKC-theta in human brain microvascular endothelium. Am J Physiol Cell Physiol 302: C1513–1522. 10.1152/ajpcell.00371.2011 22403784 PMC3362001

[4] Simi A, Tsakiri N, Wang P, Rothwell NJ (2007) Interleukin-1 and inflammatory neurodegeneration. Biochem Soc Trans 35: 1122–1126. 17956293 10.1042/BST0351122

[5] Didier N, Romero IA, Créminon C, Wijkhuisen A, Grassi J, Mabondzo A. (2004) Secretion of interleukin-1β by astrocytes mediates endothelin-1 and tumour necrosis factor-α effects on human brain microvascular endothelial cell permeability. Journal of Neurochemistry 86: 246–254. 10.1046/j.1471-4159.2003.01829.x 12807444

[6] Coisne C, Engelhardt B (2011) Tight junctions in brain barriers during central nervous system inflammation. Antioxid Redox Signal 15: 1285–1303. 10.1089/ars.2011.3929 21338320

[7] Basu A, Krady JK, Levison SW (2004) Interleukin-1: a master regulator of neuroinflammation. J Neurosci Res 78: 151–156. 15378607 10.1002/jnr.20266

[8] Blamire AM, Anthony DC, Rajagopalan B, Sibson NR, Perry VH, Styles P. (2000) Interleukin-1beta -induced changes in blood-brain barrier permeability, apparent diffusion coefficient, and cerebral blood volume in the rat brain: a magnetic resonance study. J Neurosci 20: 8153–8159. 11050138 10.1523/JNEUROSCI.20-21-08153.2000 PMC6772751

[9] de Vries HE, Blom Roosemalen MC, van Oosten M, de Boer AG, van Berkel TJ, Breimer DD (1996) The influence of cytokines on the integrity of the blood-brain barrier in vitro. Journal of neuroimmunology 64: 37–43. 8598388 10.1016/0165-5728(95)00148-4

[10] Sozen T, Tsuchiyama R, Hasegawa Y, Suzuki H, Jadhav V, Nishizawa S. (2009) Role of interleukin-1beta in early brain injury after subarachnoid hemorrhage in mice. Stroke 40: 2519–2525. 10.1161/STROKEAHA.109.549592 19461019 PMC2763121

[11] Grossetete M, Phelps J, Arko L, Yonas H, Rosenberg GA (2009) Elevation of matrix metalloproteinases 3 and 9 in cerebrospinal fluid and blood in patients with severe traumatic brain injury. Neurosurgery 65: 702–708. 10.1227/01.NEU.0000351768.11363.48 19834375 PMC2764327

[12] Chen F, Ohashi N, Li W, Eckman C, Nguyen JH (2009) Disruptions of occludin and claudin-5 in brain endothelial cells in vitro and in brains of mice with acute liver failure. Hepatology 50: 1914–1923. 10.1002/hep.23203 19821483 PMC2925168

[13] Wu B, Ma Q, Khatibi N, Chen W, Sozen T, Cheng O. (2010) Ac-YVAD-CMK Decreases Blood-Brain Barrier Degradation by Inhibiting Caspase-1 Activation of Interleukin-1beta in Intracerebral Hemorrhage Mouse Model. Transl Stroke Res 1: 57–64. 10.1007/s12975-009-0002-z 20596246 PMC2892994

[14] Wiggins-Dohlvik K, Merriman M, Shaji CA, Alluri H, Grimsley M, Davis ML et al (2014) Tumor necrosis factor-alpha disruption of brain endothelial cell barrier is mediated through matrix metalloproteinase-9. Am J Surg 208: 954–960; discussion 960. 10.1016/j.amjsurg.2014.08.014 25312844

[15] Rudra DS, Pal U, Maiti NC, Reiter RJ, Swarnakar S (2013) Melatonin inhibits matrix metalloproteinase-9 activity by binding to its active site. J Pineal Res 54: 398–405. 10.1111/jpi.12034 23330737

[16] Wiggins-Dohlvik K, Han MS, Stagg HW, Alluri H, Shaji CA, Oakley RP et al (2014) Melatonin inhibits thermal injury-induced hyperpermeability in microvascular endothelial cells. J Trauma Acute Care Surg 77: 899–905; discussion 905. 10.1097/TA.0000000000000346 25051382

[17] Chen Y, Mao H, Yang KH, Abel T, Meaney DF (2014) A modified controlled cortical impact technique to model mild traumatic brain injury mechanics in mice. Front Neurol 5: 100 10.3389/fneur.2014.00100 24994996 PMC4061598

[18] Campolo M, Ahmad A, Crupi R, Impellizzeri D, Morabito R, Esposito E et al (2013) Combination therapy with melatonin and dexamethasone in a mouse model of traumatic brain injury. J Endocrinol 217: 291–301. 10.1530/JOE-13-0022 23532863

[19] Simao F, Pagnussat AS, Seo JH, Navaratna D, Leung W, Lok J et al (2012) Pro-angiogenic effects of resveratrol in brain endothelial cells: nitric oxide-mediated regulation of vascular endothelial growth factor and metalloproteinases. J Cereb Blood Flow Metab 32: 884–895. 10.1038/jcbfm.2012.2 22314268 PMC3345913

[20] Stagg HW, Whaley JG, Tharakan B, Hunter FA, Jupiter D, Little D et al (2013) Doxycycline attenuates burn-induced microvascular hyperpermeability. J Trauma Acute Care Surg 75: 1040–1046; discussion 1046. 10.1097/TA.0b013e3182aa9c79 24256679

[21] Vermeer PD, Denker J, Estin M, Moninger TO, Keshavjee S, Karp P et al (2009) MMP9 modulates tight junction integrity and cell viability in human airway epithelia. Am J Physiol Lung Cell Mol Physiol 296: L751–762. 10.1152/ajplung.90578.2008 19270179 PMC2681350

[22] Ruhul Amin AR, Senga T, Oo ML, Thant AA, Hamaguchi M (2003) Secretion of matrix metalloproteinase-9 by the proinflammatory cytokine, IL-1beta: a role for the dual signalling pathways, Akt and Erk. Genes Cells 8: 515–523. 12786942 10.1046/j.1365-2443.2003.00652.x

[23] Breslin JW, Zhang XE, Worthylake RA, Souza-Smith FM (2015) Involvement of local lamellipodia in endothelial barrier function. PLoS One 10: e0117970 10.1371/journal.pone.0117970 25658915 PMC4320108

[24] Adderley SP, Lawrence C, Madonia E, Olubadewo JO, Breslin JW (2015) Histamine activates p38 MAP kinase and alters local lamellipodia dynamics, reducing endothelial barrier integrity and eliciting central movement of actin fibers. Am J Physiol Cell Physiol 309: C51–59. 10.1152/ajpcell.00096.2015 25948734 PMC4490326

[25] Dong L, Qiao H, Zhang X, Zhang X, Wang C, et al (2013) Parthenolide is neuroprotective in rat experimental stroke model: downregulating NF-kappaB, phospho-p38MAPK, and caspase-1 and ameliorating BBB permeability. Mediators Inflamm 2013: 370804 10.1155/2013/370804 23935248 PMC3725704

[26] Asahi M, Asahi K, Jung JC, del Zoppo GJ, Fini ME, Lo EH. (2000) Role for Matrix Metalloproteinase 9 After Focal Cerebral Ischemia: Effects of Gene Knockout and Enzyme Inhibition With BB-94 J Cereb Blood Flow Metab 20: 1681–1689. 11129784 10.1097/00004647-200012000-00007

[27] Seifman MA, Adamides AA, Nguyen PN, Vallance SA, Cooper DJ, et al (2008) Endogenous melatonin increases in cerebrospinal fluid of patients after severe traumatic brain injury and correlates with oxidative stress and metabolic disarray. J Cereb Blood Flow Metab 28: 684–696. 10.1038/sj.jcbfm.9600603 18183032

[28] Dehghan F, Khaksari Hadad M, Asadikram G, Najafipour H, Shahrokhi N (2013) Effect of melatonin on intracranial pressure and brain edema following traumatic brain injury: role of oxidative stresses. Arch Med Res 44: 251–258. 10.1016/j.arcmed.2013.04.002 23608674

