# Supplementary figures and images for: Melatonin Preserves Blood-Brain Barrier Integrity and Permeability via Matrix Metalloproteinase-9 Inhibition
Source: PLoS One. 2016 May 6;11(5):e0154427. doi: 10.1371/journal.pone.0154427 (PMC4859489; doi:10.1371/journal.pone.0154427)

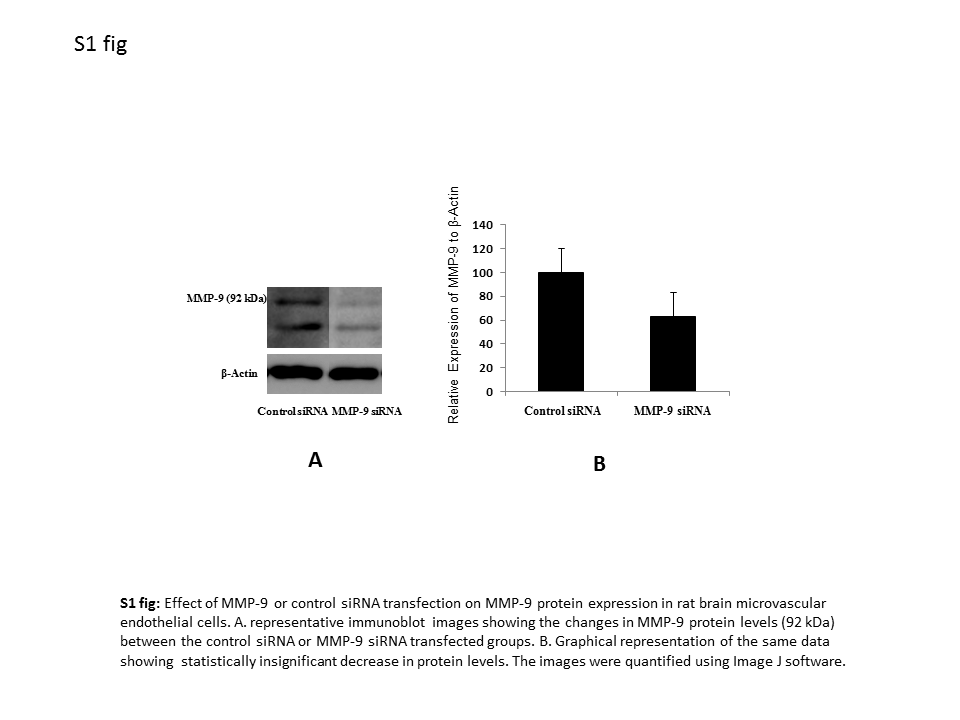

Supplement: S1 Fig — A. Representative bands from immunoblot analysis. B. Analysis of the data using ImageJ (Student’s t-test; p>0.05). The data is expressed as relative expression of MMP-9 to β-Actin. (TIF) [file pone.0154427.s001.tif]
